# Editorial Introduction: The Shifting Geopolitics of Return Migration and Reintegration

**DOI:** 10.1007/s12134-022-00974-x

**Published:** 2022-06-27

**Authors:** Zana Vathi, Russell King, Barak Kalir

**Affiliations:** 1 Edge Hill University, Ormskirk, UK; 2 University of Sussex, Brighton, UK; 3 University of Amsterdam, Amsterdam, The Netherlands

**Keywords:** Return migration, Geopolitics, Migration governance, Ontological (in)security, Reintegration

## Abstract

The recent geo-politicisation of return migration warrants deep consideration of the politics of return and reintegration. A focus on geopolitics prefigures the study of reintegration not just as circumstantial to the lives of migrants or the formal strategies of states but also as deeply embedded in the historical socio-cultural and political contexts where it takes place. In introducing a set of papers that explore these links from different angles and based on research from around the world, this article argues that return and reintegration constitute a qualitatively different process from immigration and integration in the receiving countries, first and foremost because the sending state—a key actor in the reintegration process—is in a position of geopolitical power marginality. Indeed, the strategies of all the stakeholders implicated in reintegration are closely linked to the geopolitics of migration governance. In these contexts, migrants’ intimate, as well as pragmatic, strategies of reintegration and re-migration are an expression, as well as a trigger, of multi-scale geopolitics. There is a distinct contrast between the emphasis on borders and securitisation in high-income countries and the informality and precarity of the way that migrants have to manage their ontological security in the process of return and reintegration. Reintegration should thus be understood as a process contingent upon different and, often, incongruous legal, political and socio-economic elements, as endorsed and employed by the different stakeholders involved.

## Introduction

The symbolic and instrumental positioning of return migration within public discourse and migration agendas at both national and international levels has changed significantly in recent years. Multiple forms and framings of crises, such as financial, environmental and refugee crises and, most recently, the COVID-19 crisis, appear closely linked with this transformation. Evidence suggests that return can be leveraged to serve nationalistic political projects as well as international geopolitical goals (Cuttitta, 2018). A highly sensitive issue, return often requires complex operations and supplementary resources to be mobilised and accomplished, a good example being the 2016 EU–Turkey agreement. In many contexts, some forms of forced return are appearing as assaults on human rights, such as the deportation of the Windrush immigrants from the UK (April 2018) or the recent forced return of migrants from Saudi Arabia to Ethiopia in November 2020.

The countries of origin to which migrants return are often in a precarious bargaining position when it comes to negotiating the terms for processing the migrants’ return or managing their resettlement and reintegration. Their poorly resourced social-protection system is often one of the reasons why migrants left in the first place (Vathi et al., 2019). Often, volatile economic politics in these countries lead to reversing trends in migration and return waves; the recent returns of Colombians from Venezuela due to the economic crisis that has plagued the latter have been followed by the immigration of Venezuelans to Colombia, which has had a negative impact on poverty outcomes for native Colombians (Caruso et al., 2021; see also the paper by Riaño in this special issue). In the Global South, return is little researched, even though there is growing evidence of the interconnectedness between (re-)migration patterns in these contexts and those in high-income countries (Dünnwald, 2017). At the same time, different countries in the Global South as well as in the Middle East are emerging as gravity points for patterns of regional migration and return.

However, even though migration is considered a barometer of or even a medium for geopolitics (Hyndman, 2012), there is little interest in and understanding of the extent to which return migration is linked to geopolitics; it is the purpose of this special issue to rectify this. For a long time, return migration was seen within the framework of sustainability and the more intimate aspects of migrants’ lives were overlooked. When introducing a feminist geopolitical lens, it becomes apparent that the way in which power influences return is complex and has ramifications for social and political relations at different scales (cf. Dowler & Sharp, 2001; Setrana & Tonah, 2014; Williams & Massaro, 2013). Power is thus an important factor of the way in which return is conceptualised, regulated and experienced in both public and intimate spheres.

We need, firstly, to acknowledge the problematic definition and construction of the term ‘return migration’ (King, 2000). Too often it is construed and naturalised as a teleological move of migrants back to the place ‘where they truly belong’. This formulation is mistaken on several counts. First, by virtue of their multilocal migration experience, migrants may feel a ‘belonging’ to two or more places, not just to their ‘home’ country. Second, there can be no true ‘return’ to the *status quo ante*: In their absence, the countries of migrants’ origins will have changed, perhaps beyond recognition. Moreover, migrants, too, will have changed by virtue of their migration experience and life-stage maturation. And the people ‘back home’ will likewise have changed, so that old friendships and relationships cannot always be resuscitated, at least not on the terms prior to migration (Vathi et al., 2019). Third, the notion of return as the final closure of the migration cycle needs to be questioned. There are many different temporalities of return (for visits, for shorter or longer periods of sojourn or for good) and return may just be an interim stage in a lifetime of transnational mobility involving multiple locations (Bilgili, 2022; King & Christou, 2011).

Perhaps due to the complexity of return migration, our current understanding of reintegration remains vague (Marino & Lietaert, 2022). Indeed, this lack of a clear conceptual framework goes in tandem with the symbolic and instrumental positioning of return and reintegration within public discourse and migration governance (IOM, 2017). Four main points are observable in the limited existing literature and ongoing policy-making. Firstly, the major geopolitical dimensions that underpin migration and return are often treated separately—both politically and academically—from the reintegration processes on the ground. Secondly, the meaning attached to reintegration differs significantly between policy-makers, researchers and returnees (Lietaert, 2016). Thirdly, the attitudes towards and experiences of reintegration processes vary across contexts—notably by age, gender, modes and motives of return, social networks and family composition (Vathi, 2017). Fourthly, in the context of ‘voluntary’ returns, empirical research shows that migrants experience a variety of post-return mobilities, whilst they themselves are progressively changed by both migration abroad and relocation to the country of origin. Furthermore, return is often used as a proxy for deportation and other forms of forced return, even when it is referred to by state officials and civil-society actors as ‘voluntary’ (Kalir, 2017, 2022). These different connotations are important, as the conceptualisation of return has implications for the support offered to migrants experiencing relocation to the country of origin through different means—such as removal, deportation, repatriation or assisted voluntary return. This has led recent scholarship to coin the term ‘re-entry’ to accommodate the diversity of migrant scenarios (Lietaert & Van Gorp, 2019).

This special issue aims, then, to address the multi-dimensional nature of reintegration and the contradictions between ideological positions, policies and practices in relation to it. It does so by establishing links between geopolitical shifts at different scales, their impact on return and the viability, dimensions and ‘outcomes’ of reintegration. For the purpose of this special issue, we define geopolitics as ‘the nexus – created in terminology and practice of geopolitics – between society, space and power; the nexus which is not essentially pre-existent, but that forms a fragment of the hegemonic discourses of modernity and has a powerful impact on social practice’ (Reuber, 2009: 451). Feminist takes on geopolitics are particularly pertinent to this special issue in the way that they ‘link international representation to the geographies of everyday life; [and help to] understand the ways in which the national and international are reproduced in the mundane practices we take for granted’ (Dowler & Sharp, 2001: 171).

The papers which follow are based on rich empirical research on the ground—both qualitative and quantitative—and address critical aspects of return migration which, otherwise, are to date unexplored. The diverse contributions cover geographical contexts across five continents and a much higher number of migration and return contexts around the world, with some papers based on ambitious comparative designs of two or more countries. Most importantly, the contributions shift the focus from the Global North and high-income countries in the West, to different gravity points across the globe where migration and return are intertwined with important bi-country and multi-country geopolitical implications.

## Return and Reintegration from a Feminist Geopolitical Stance: The Missing View?

Cross-border movements are closely linked to geopolitics; they can be an expression or a trigger of geopolitical dynamics. However, it is a long time since international migration and even refugee flows have been the central focus of scholars of critical geopolitics (Allen et al., 2018; Tesfahuney, 1998). Instead, a general lack of focus on the reproduction of—and resistance to—geopolitical relations is observable (Sprake, 2000) and, in the case of return migration, this is particularly evident and largely unwarranted.

The lack of direct focus on geopolitics and migration is countered to some extent by research that looks at top-down migration governance (Sahin-Mentucek et al., 2022), the mundane experiences of mobility and the strategies of migrants against regimes of migration control (Papadopoulos & Tsianos, 2013).

When it comes to return migration, the link with geopolitics is obscure because of an overwhelming conception of migration as linear and of return migration as ‘homecoming’ (Markowitz & Stefansson, 2004; Taylor, 2017). An assumption also exists on the part of high-income countries that their responsibility towards migrants ceases when the latter leave their territory, thus making geopolitics of return seemingly trivial for migration scholars. However, the long-standing emphasis on the sustainability of return, which has evolved into the sustainability of reintegration (IOM, 2017), is well within the realm of geopolitics—something which the papers in this special issue investigate from different angles.

The governance of return and its geopolitics are transnational in nature, involving sending, transit and receiving countries. Whilst this may have been the case in the past—for example, EU readmission agreements with the so-called third countries—there has been a more sustained geo-politicisation of return since 2015 due to the so-framed ‘refugee crisis’ (Cuttitta, 2018). This geo-politicisation indicates a reactive approach to managing migratory flows as well as a proactive one, with the key purpose of deterring future migration. A political agenda based on populist calls for the reduction of migration rates and strong economic imperatives in general—and the repercussions of neoliberalism, more particularly—underpins these approaches to return and the specific return schemes employed in high-income countries. This agenda is ironically legitimised by linking it with the promise of stronger protection for asylum-seekers’ rights (Triandafyllidou & Ricard-Guay, 2019). Increasing pressure on enforcing EU return policy is believed to lead to more deportations, which increases migrants’ precarity upon return.

The EU readmission agreement scheme has evolved as part of the recent New Pact on Migration (European Commission, 2020). Indeed, the whole set of schemes of ‘voluntary return’ enshrines the unequal geopolitics of return (Lietaert, 2022). They appear to embody and further trigger the political bargaining of the different stakeholders—states, service-providers, migrants, communities and families in the country of origin, markets and employers (Flahaux, 2020) —for recognition and assertion of their positionality in the process and power politics at different scales. As Ashutosh and Mountz (2012: 352) maintain, migrant journeys are ‘shot through with spatial impositions of power on mobility and the human agency of individuals and communities navigating geopolitical hierarchies’. How this spatial imposition of power applies in the context of return migration has been very little researched.

The recent geo-politicisation of return migration can be seen within the broad framework of migration and border management. It reflects upon the increase in the regulation of the relationships of the EU with third countries as part of the broader securitisation agenda (Cassarino, 2016). The EU–Turkey agreement in 2016 and the subsequent EU and bilateral policies are based on the principle that the politics of border control, as central to migration regimes, need two states as parties—the receiving and sending states. However, these policies and actions remain significantly unequal. First, they pay little attention to migrants’ human rights, let alone their complex migration motivations; secondly, they are often, in reality, the imposition on the third country of a ‘partnership’ with the EU, whilst there is hardly any consideration of the interests of the former. Indeed, these partnerships can lead to further strain on the local and regional socio-economic situation in the third countries involved (see Triandafyllidou & Ricard-Guay, 2019).

Countries of origin that become receiving countries for returnees have employed a variety of reintegration strategies, following a series of ineffective ad hoc measures (Arowolo, 2000). Often these national reintegration strategies take place under the pressure of macro-level geopolitics, such as accession into the EU for the Western Balkans, for example. The way in which these policies are implemented leaves much to be desired, due to the lack of coordination between the different agencies and the overall poor infrastructure. Often the pressure on the countries of origin to admit returnees is not accompanied by support for their reintegration on the ground. A transnational gap therefore emerges between the high-income countries that return migrants and the impoverished origin countries that are expected to provide reintegration services for their returning citizens. Recent attempts to fill this gap are observable, calling for continued cooperation between high-income countries and countries of origin in programme design and implementation; however, the effectiveness of these initiatives is still largely unknown (OECD, 2020).

How do return migrants and their families make sense of and react to such unequal macro-level geopolitics? How do these geopolitics apply in the context of return and reintegration in lower-income countries, in particular in the Global South, where different degrees of (in)formality are part of governance regimes and interpersonal relations? These questions are particularly important when considering that alongside the geo-politicisation of return migration goes the growing conceptualisation of it as the removal of irregular migrants from high-income countries (Kalir, 2019; Triandafyllidou & Ricard-Guay, 2019). These geopolitics create hierarchies of categories within the return-migrant population, leaving out of the focus of scholars and policy-makers those migrants whose return is not implicated in the removal and assistance schemes. With the main aim being the translation of macro-level geopolitics into local and regional systems of sustainable return, the purpose of these schemes is to further legitimise the unequal migration-management regimes in place. They also contribute to the different political weights of the specific categories of returnee, as deeply embedded in the broader geopolitics of cross-border movements (Allen et al., 2018).

The way that migrants engage in these politics, by either instrumentally utilising AVRR (Assisted Voluntary Return and Reintegration) schemes or resisting them, is helpful in analysing ‘doing geopolitics’. This also includes migrants’ indifference towards or even lack of awareness about these schemes (Vathi et al., 2019), which should be seen within the framework of ‘geopolitics from below’. To date, a rather state-focused definition of geopolitics has been employed in the analysis of state-sponsored return schemes. However, in the context of migration, closer attention should be paid to the analyses that build upon close readings of specific groups from particular historicised places that create new grounds for ‘doing geopolitics’ (Hyndman, 2012: 253). Therefore, despite the significant role of the state in legitimising some forms of cross-border mobility whilst controlling its borders, cross-border movements that are part of return, reintegration and/or re-migration warrant a more sophisticated take on geopolitics or on the nexus between society, space and power (Reuber, 2009).

Reintegration can thus only be explored thoroughly through a critical geopolitical lens. Even though reintegration upon return is now an established concept in migration scholarship, the processes it encompasses have mostly been studied indirectly within the framework of the sustainability of return. The latter is, in itself, a concept testifying to the unequal geopolitics of return, as it refers to the goals of persuading returnees to stay put upon return for as long as possible, regardless of the context of return or their personal circumstances. Over time, the study of reintegration has benefitted from advances in research on transnationalism and social networks and, more recently, psychosocial well-being (Vathi, 2022). A growing awareness recognises reintegration as a challenging, multi-dimensional and highly contextualised process (Black & Castaldo, 2009; Carling & Erdal, 2014; Markowitz & Stefansson, 2004). In particular, the characteristics of the returnees, the modes of return and the conditions in the host country and the country of return are identified as key factors that affect reintegration (Cassarino, 2004; Kuschminder, 2017).

Yet, the geopolitical aspects of reintegration are somewhat overlooked in return-migration scholarship (Ho, 2012). The take on this process as contingent on the type of return and with the purpose of establishing whether returnees will contribute to the development of the country of origin or not is already an endorsement of the Global North agendas on governing migration and return which underpin the AVRR schemes. By researching, analysing and re-conceptualising reintegration through the lens of feminist geopolitics (Williams & Massaro, 2013), with a focus on migrants’ experiences and their own positionalities towards migration and return regimes, a deeper understanding is gained on why and how returnees adopt (or not) certain strategies of preparedness prior to return (Cassarino, 2014), as well as adaptation, settlement or re-migration following return. In other words, by engaging with geopolitics at different scales, reintegration is studied beyond the goals of the Global North’s agendas, although it is as deeply embedded in the socio-political contexts where it takes place.

## Introducing the Contributions: the Multi-scalar Links Between Return, Reintegration and Geopolitics

This set of papers approaches reintegration not only as a process that unfolds on the ground but also as a stage in diverse and evolving migration trajectories; a process which is affected by and, to a certain extent, affects geopolitics at different scales. The interplay of micro-level and meso-level dynamics is particularly overlooked in research on return (Parella & Petroff, 2019), whilst reintegration has rarely been linked with macro-level geopolitics. Shifting the focus on to lower-income countries in Europe (e.g. Hungary, Latvia, the Western Balkans) and the Global South (e.g. Colombia, Ghana, Cameroon) follows a recent call to move away from Eurocentricity or the centrality of the EU legal and logistical framework on migration management when analysing migration governance (Triandafyllidou & Ricard-Guay, 2019).

The very status of returnees and the ‘returnee industry’ as part of AVRR schemes is problematised by Shaidrova, whilst new concepts are coined such as *forced transmobilities*, as demonstrated by Riaño, and *cognitive remittances*, introduced by Vathi, King and Gëdeshi in their paper. Key concepts of return-migration research are applied in several papers to different groups of returnees, such as the *preparedness* of returnee children (Grosa & King), the *stigma* of highly skilled professional returnees who engage in *return visits* (Mueller & Kuschminder) and *mentorship schemes* upon return (Majidi et al.). New light is shed on certain migration and return contexts which have previously attracted less attention (e.g. Lados et al. on intra-EU returns) or the study of which has lacked focus on returnees’ own aspirations (e.g. Guzmán Elizalde’s paper on Mexico–US cross border movements and well-being) and the return of ethnic minorities to their country of citizenship (e.g. the Roma, see Vathi et al.). The crucial role of the family in the entire migration and return trajectory in a Global South setting—Cameroon—is revisited by Wanki, Lietaert and Derlyun, not only in terms of its emotional and instrumental aspects but also as a key factor in reintegration, in tandem with complex geopolitics that surround the different forms of return migration. The losses that migrants incur are also discussed by Mingot and Rudolph, with a view to identifying what needs re-establishing upon return for a less-bumpy reintegration.

The papers in this collection can be broadly grouped under three main interrelated themes: (1) macro-level geopolitical dynamics and returnees’ positionality (Shaidrova; Majidi et al.; Mingot & Rudolph); (2) structural dimensions of reintegration and their sensitivity to regional, national and local geopolitics (Vathi et al.; Riaño; Mueller & Kuschminder; Grosa & King); and (3) returnees’ agency, community responses and the more intimate effects of geopolitics on return and reintegration (Lados et al.; Guzmán Elizalde; Wanki et al.). Let us now expand on each of these crucial themes.

### Returnees’ Positionality Towards Macro-level Geopolitics

Papers that are primarily focused on *macro-level geopolitics* are broadly concerned with unpacking states’ and macro-level actors’ philosophies of return and the positionality of the different state and non-state actors across space and time, whilst considering the influence of specific contexts. Employing a feminist perspective to geopolitics, they go beyond the standard return and reintegration discourses to trace and analyse practices on the ground, showing how these discourses materialise in lower-scale unequal relations shot through with power differentials. More specifically, they look at unequal, yet intimate, negotiations of returnee status (Shaidrova), mentor and returnee relationships at the local level (Majidi et al.) and the positionality of returnees towards return-migration regimes based on their life stories and personal circumstances (Mingot and Rudolph).

As Shaidrova observes, state-funded programmes have important implications in (re)producing and shaping the ‘returnee industry’ by ‘doing’ returnee assistance and transforming repatriated migrants through AVRR into ‘returnees’. Her article is in line with critical inquiries in the context of new geopolitics of migration governance into how categories of migration are constructed and by whom and how they are challenged (Allen et al., 2018). Research has long documented the stigma that returnees face when they fail to generate substantial resources for their families (Schuster & Majidi, 2015); however, the politicised ‘returnee’ label appears to have its silver linings.

Engaging with the ‘returnee industry’ enables the returnees to counter this stigma, not least because of the opportunities that the returnee label offers for access to resources as part of the home country’s reintegration assistance. Ultimately, the (in)accessibility of a returnee status by those who do not engage with AVRR programmes is underpinned by the geopolitics of inequalities between the Global North and the Global South. States in the Global North not only control migration and return in and from their territories but the AVRR programmes also create hierarchies and inequalities within the returnee group in the countries of the Global South, with direct consequences for reintegration. The hierarchies imposed geopolitically on lower-income countries of origin are a continuation of the politics of race and racialisation of migration that underpin the urge to remove as many migrants and asylum-seekers as possible from higher-income territories. The strategies of migrants to integrate themselves in the returnee industry appear to be a particular form of resistance to geopolitical relations, a focus which is largely missing in critical geopolitical studies (Sprake, 2000).

Some of these inequalities can be reduced or even countered by the mentorship schemes which are integrated into some AVRR programmes. In their paper, Majidi et al. show that mentoring has a small but positive and significant impact on reintegration. Thus, a further expansion of approaches at the cross section of social work and reintegration may result in improved reintegration outcomes through the changing of geopolitical power imbalances in reintegration programming. Zooming in on the provision of reintegration assistance at the local level allows for the application of humanitarian concerns of return policies by focusing on returnees’ rebuilding of their lives, by enhancing their networks and facilitating the logistics of reintegration on the ground.

Whilst the migration-management regimes of the Global North are highly politicised and underpinned by politics of race and racialisation, reintegration through mentorship takes a person-centred approach, with psychosocial well-being at its core (Vathi, 2022). If the humanitarian discourse on return and reintegration has proved ineffective for returnees, not least because of it being part of a security-humanitarianism logic (Lloyd et al., 2017), the mentorship scheme appears as an effective instrument for advancing a more-humane geopolitics of reintegration.

In another context in the Global South—Ghana—and with returnees from the USA, Libya and EU countries, Mingot and Rudolph examine the implementation of AVRR programmes, taking an evaluation approach that focuses on bottom-up perspectives. They explore the range of experiences of ‘voluntary’ return to Ghana, based on the different positionalities of migrants against migration and return regimes and broader socio-economic and racial inequalities. Their investigation is embedded in the analysis of the ways that Ghana’s return policy reflects its geopolitical orientations. This examination illustrates how geopolitical relations shape migrants’ mobilities, highlighting the unequal and evolving relations between the different actors in the countries of origin and destination—primarily the migrants themselves, their relatives and communities of origin and the policies on return.

A lack of consensus between, as much as the differing interests and expectations among, these actors creates unequal options for and expectations of mobility. Therefore, migrant journeys, regardless of age, gender, legal status, social class or race, are always geopolitical journeys, even though the way that geopolitics affects each individual migrant depends on the intersection of these key characteristics. The different ideas and experiences of return depend not only on the individual situations but also on the broader politicised relations and interests between stakeholders in the migration and return processes. The inequalities that underpin these relations are also at the basis of losses intrinsically linked to migration and return. Not all migration journeys benefit migrants; therefore, reintegration policies and programmes should identify those elements that have to be re-established to achieve an embedded reintegration.

### Structural Dimensions of Reintegration and Regional, National and Local Geopolitics

This set of papers is concerned with the role of *policy and institutional practices* and shifts in migrants’ perspectives. The articles analyse how these shifts affect attitudes and behaviours in the return process, as structural aspects of contexts where return takes place are linked to historical and ongoing geopolitical dynamics.

One underexplored setting, particularly from the perspective of highly skilled returnees—the institutional setting—is analysed by Mueller and Kuschminder, who focus on short-term reintegration and stigma across three African countries: Ethiopia, Sierra Leone and Somalia. Based on identity politics as well as a transactional perspective, this paper explores how personal and institutional positionality reflects the expectations of highly skilled diasporans prior to return, their experiences of stigma during return and the strategies they have developed to counteract stigma. The findings show that stigma in the case of highly skilled returnees for knowledge transfer is rooted in the perceived inequalities between home-country employees and communities of origin in comparison to these returnees. Home-country employers, state actors and their inter-relations make up the backdrop against which these meso-level geopolitics pan out.

These inequalities are broadly underpinned by global inequalities in terms of citizenship and access to international professional mobility. These findings add to our understanding of reintegration—how temporary returns and stigma management can be seen as an astute strategy of ‘fitting in’ without countering the conventions of professional settings. They show how an advantageous positioning of returnees in terms of power hierarchies and access to international mobility or to ‘enabling citizenship’ (Vathi, 2017) can provide superficial endorsement and acceptance of the differing work ethics on the part of returnees. Reintegration behaviour, therefore, can be pragmatic and socially astute but only when the geopolitics of international mobility are on the returnee’s side.

Integrating the structural with the informal, Riaño looks at the role of business ventures in enabling or enhancing the reintegration of migrants in Colombia who are returning from neighbouring Venezuela, in her analysis of *forced transmobilities*. Returnees as ‘agents of development’ refers to, firstly, a politicised notion of migrants’ capital, which is usually discursively employed in return agendas in countries of destination and targeted in the context of return by countries of origin. However, despite remarkable resilience, commitment, creativeness and personal resources in the form of skills and family support, the vast majority of returnees in Colombia struggle to reintegrate and to create sustainable businesses that satisfy their fundamental, existential and safety needs. The forms of migration and the migration contexts concerned play an important role in promoting or obstructing both transnational ties and the level and quality of the capital which migrants accumulate and can deploy post-return.

International and local geopolitics appear to impact on the transmobilities of returnees as well as their reintegration processes. There seems to be no formal accountability for the harsh politics and practices of return/deportation in this South–South context and a sense of insecurity trickles down to the most intimate spheres of migrants’ lives, including their sense of personal security. The role of entrepreneurship in reintegration is thus questioned, in view of the unequal opportunities that the local context offers to returnees. Instead, migrant entrepreneurship appears here both as a vehicle with which returnees can deploy their financial and social remittances and as a fulcrum in which the geopolitics of South–South cross-border translocal movements are exemplified in their material and logistic precarities.

However, issues of insecurity are also found elsewhere, such as in the way that age places young returnees in a position of political inferiority and subjects them to certain national agendas. In Latvia, returnee children face multi-dimensional geopolitics that underpin their structural experiences of schooling. Grosa and King capture these by utilising a multi-stakeholder analysis involving children, parents and teachers. This post-communist Baltic state, which became an EU country in 2004, offers a rich context for the analysis of how children experience the geopolitical dimensions of their context of reintegration through teachers’ attitudes and the ideology of the education system (see also Grosa, 2022; Vathi et al., 2018). The key contribution of their paper is its evidence on the importance of the preparedness (Cassarino, 2004) of ‘returnee’ children and the role of schools in reintegration. Due to the educational challenges that returning children face, tackling these two in conjunction would enhance children’s psychosocial well-being and educational performance. The findings show that children’s preparedness is contingent upon their transnational perceptions of the two places and their (lack of) expectations, the parents’ engagement in presenting return as a gain to the children as a way of involving them in their decision-making and the instrumental role of language classes held at weekends before embarking on return journeys. Therefore, school systems and the differences that returnee children experience are part of geopolitical shifts in the return-migration process, as they are based on different ideologies and histories for each country, which are ingrained and further enhanced by the education system. In turn, macro-level geopolitics such as EU integration and conditionality towards the newer members lead to the mainstreaming of the education system with EU standards. This geopolitical realignment of the education ideology impacts on the experience of ‘diverse’ children in schools.

The EU and intra-European migrations are also the focus of Vathi et al.’s paper, this time in the context of returning ethnic minorities. Vathi and her colleagues analyse the experiences of Roma returnees relocating to Albania and Kosovo, noting a significant shift in their perspective on their positionality in these countries, whilst these countries’ EU accession process becomes the complex political background against which these returns are considered in policy-making. The consequences of politicised identity markers for migrants, such as race and ethnicity, religion and gender, have been discussed in the migration literature in the context of new geopolitics of migration governance (Allen et al., 2018) but very little in the context of return. The particular experiences of the Roma testify to the importance of social capital in giving rise to *cognitive remittances* among these returnees, with implications for both their reintegration and their re-migration tendencies. These cognitive remittances consist of the individual reappraisal of an individual’s group political identity that is triggered by the observation of the different status of the group across different socio-political systems. In other words, the distinctly discriminated-against Roma in the Western Balkans sense their place in the socio-political hierarchy is not as disadvantageous in EU countries and this leaves a blueprint on their self-perception. It further translates into different attitudes towards the socio-political system in the country of origin in the Balkans, making them aware of and reluctant to accept the status quo.

Therefore, the multi-scale geopolitics of the Roma return is first linked to the change in status of the Western Balkan countries by the EU into ‘safe countries of origin’, which doomed their asylum-seeker applications. Nevertheless, return itself makes the Roma into agents in geopolitics since their cognitive repositioning against a social structure that put them right at the bottom for centuries and their strategies to counter this show their engagement at the intersection between society, space and power (Reuber, 2009). Therefore, geopolitics of return need not be about state borders; they can also be about how international mobility emancipates a person’s perception of his or her political self and reorders the perception on the hierarchy of belongingness and strategies around it.

### Intimate Effects of the Geopolitics of Return and Re-integration

Returnees’ agency, community responses and *the more intimate effects of geopolitics of return and re-integration* take centre-stage in the papers by Guzmán Elizalde, Lados et al. and Wanki et al. Return can be an open-ended process, according to Guzmán Elizalde, who integrates the metropolitan context of Mexico City with that of a rural setting in the state of Puebla to analyse the ‘outcomes’ of return for Mexican migrants. Re-migration appears as a strategy to ensure the ideal of *vivir mejor* (living better), whilst the inability to do so generates significant challenges for reintegration. The findings link, in this way, reintegration with the geopolitics of well-being along the Mexico–USA migration corridor which, in terms of the scale of movement, is the largest bilateral migration flow in the world. The majority of the participants appear to return for personal reasons and constraints linked to the life course, such as the end of a relationship or care for the elderly left behind, whilst facing a different reception in the capital city when compared to provincial towns. The diverse profile of return scenarios, not least the very emotional and intimate nature of the reasons for return for many and the difference in reception by the communities of origin, all have implications for the way that policies are designed and, even more importantly, how they are implemented on the ground. Whilst return can be leveraged to serve specific geopolitical goals, policies should offer sustained instrumental (material assistance), informative (advice and guidance) and psychosocial support throughout return migrants’ lengthy process of reintegration.

A similar approach is taken by Lados et al., who touch upon the geopolitics of intra-EU migration—a process that has intensified in the COVID-19 era—through the lens of ontological (in)security (Giddens, 1991). For quite some time, this type of migration has been considered free from geopolitical tensions; EU citizenship affords extensive rights for mobility and employment and, to some extent, for social protection across EU member countries. Return migration in this context is very little researched, not least because movements in the EU space are mostly analysed through the framework of mobility (Favell, 2016). However, economic and political inequalities within the EU are well known and inevitably affect the direction of population movements, the experiences of Central and Eastern European migrants in the West (Ciupijus, 2011) and their tendency for return migration.

Yet these processes are different from the migration and return of migrants in other parts of the world, not least because of the different geopolitics that underpin them. Lados et al. go beyond the discussion of intra-EU free movement when analysing the migration and return of Hungarian migrants. Framing their analysis on the concept of ontological (in)security, they discuss the experiences of high-skilled and low-skilled migrants throughout the migration and return cycle. Migration and return appear to have ontological (in)security—the (in)security of the self (Giddens, 1991)—as an underlying factor. However, this displays itself differently in the motivations of the high-skilled and low-skilled migrants who expectedly also differ in terms of their identification with the culture of origin. The return programmes of Hungary appear to engage geopolitically with this search for ontological security to attract highly skilled returnees in the way that they frame the advantages of returning home, which adds another somewhat unexplored dimension to this special issue. The COVID-19 pandemic therefore poses an opportunity for the countries of origin to retain returnees with their financial and social capital through their diaspora-return policies.

In their analysis of returnees’ families and their role in return and reintegration, Wanki et al. engage in a complex analysis of the more intimate aspects of family geopolitics in the context of Cameroon. Pooling together returnees with differing degrees of freedom to return and varying levels of resources, they note that a family’s consent to return is important, both during the preparation for an impending return, following the return and in the process of reintegration. As this consent depends on the resources that returnees take back with them, reintegration is bound to pan out very differently for the different groups of assisted returnees, deportees and voluntary returnees. These differences open up a debate on the role of geopolitics at different scales that underpin return policies and programmes. This disparity manifests itself in the social support which returnees receive, which is crucial in their multi-phased and non-linear reintegration process (Lietaert & Kuschminder, 2021).

Employing a concept of ‘families’—which includes their immediate and extended families and, in some cases, their friends—Wanki et al.’s study unveils the role of the immediate and extended families and the incompatibility of their agendas and expectations with states’ geopolitics of return. The meaning and role of families are revealed as powerful units where mobilities are envisaged as a collective project of livelihood. This is against the logic of migration and border management in the higher-income countries where migrants seek opportunities to realise these livelihood projects, which aim to curtail migrants’ rights and control and reduce migration routes to a minimum.

## Concluding Discussion

The papers in this special issue show that, as returnees continue to emerge as a population that should be controlled and streamlined with political agendas, the governance and practices of return and reintegration are not independent of geopolitics. Firstly, the involvement of higher-income countries in return governance—be it through politics of border management that invoke return as a penalty or, more directly, through AVRR schemes—is making return migration a highly ‘disparate affair’ (Hyndman, 2012: 245). Reintegration assistance, for example, is linked to macro-level geopolitics around the subjects of return governance, involving locally based stakeholders in a process of delegating soft power. Secondly, unlike the context of integration in receiving countries, the role of the nation-state is less invoked by migrants in the process of reintegration, not least because of their disbelief in the power of these states (see also Vathi et al., 2019). The key dynamics of reintegration lie with other stakeholders—service-providers, communities and families of origin, employers and markets—and depend on the ways that they are positioned against global and regional migration governance, which are complex and impactful.

There is thus a contrast between the emphasis in the formal agendas of migration management on borders and securitisation in high-income countries and the informality and precarity of the way that migrants have to manage their ontological security in the process of return and reintegration. When applied to the return context, the concept of ontological security (Giddens, 1991)—a goal of preserving the self in addition to maintaining physical security—appears to encompass the public and intimate sphere of the variety of strategies that returning migrants employ against constraining geopolitics of return and less-than-ideal contexts of reintegration. This sense of personal security is cultivated through and further deployed in reintegration behaviour, which often involves returnees astutely navigating cultural repertoires and moral expectations, as exemplified by the strategies of highly skilled returnees or many others negotiating family and kin expectations. This is not least because of the prominence of the family in its broad sense in the countries where return takes place, making family an important site (Botterill et al., 2020) through which the geopolitics of return migration operate and processes of reintegration unfold.

This intimate sphere is nonetheless not immune to the geopolitical shocks and pressures operating at the international and national level, not least because of the weak social-protection systems and the overall inferior positioning of migrants’ countries of origin in the rapidly shifting global geopolitics. The case of migrant entrepreneurship upon return exemplifies the complex impact of weak social protection systems, first pushing returnees towards self-employment and, secondly, by enhancing the material and logistic precarities that returnees face when trying to establish a livelihood on their own.

However, the intimate versus the public is yet another dynamic but little explored nexus in return migration. Engaging successfully with strategies that ensure ontological security is prevalent in migrants who have a high degree of control over their journeys—those who are positively positioned against the macro-level geopolitics of cross-border movements, such as highly skilled or intra-EU migrants. Even in marginal cases, such as that of Roma returnees, international mobility and political emancipation on their discriminated-against minority status shape their reintegration behaviour. For others, *losses* as part of the migration process permeate the reintegration process, often alongside imagining, planning or engaging in re-migration.

The contributions in this collection of articles, however, portray migrants as skilled agents of bottom-up geopolitics who, even when they lack the capacity to fully decipher the geopolitics of migration governance, are able to utilise their migration capital to position themselves as favourably as possible against socio-political regimes of value, morality and extra-territorial belongingness and governance in the context of return. This is an emancipating finding, for scholars and policy-makers alike, since much of the reintegration literature either focuses on the effectiveness of return programmes or sees reintegration as an eventual process undertaken by willing and geopolitically naïve returnees.

Reintegration should, instead, be understood as a process contingent upon different and often incongruous legal, political and socio-economic elements, as endorsed and employed by the different stakeholders involved. The effects of the COVID-19 pandemic on intensifying return have undoubtedly created new dynamics, as well as research opportunities, for exploring the multi-scale geopolitics of reintegration—both the strategies of the countries of origin to retain returnees as well as the new ontologies of (im)mobility, with their political and mundane forms.
